# Dance Type and Flight Parameters Are Associated with Different Mushroom Body Neural Activities in Worker Honeybee Brains

**DOI:** 10.1371/journal.pone.0019301

**Published:** 2011-04-26

**Authors:** Taketoshi Kiya, Takeo Kubo

**Affiliations:** Graduate School of Science, Department of Biological Sciences, The University of Tokyo, Tokyo, Japan; University of Sussex, United Kingdom

## Abstract

**Background:**

Honeybee foragers can transmit the information concerning the location of food sources to their nestmates using dance communication. We previously used a novel immediate early gene, termed *kakusei*, to demonstrate that the neural activity of a specific mushroom body (MB) neuron subtype is preferentially enhanced in the forager brain. The sensory information related to this MB neuron activity, however, remained unclear.

**Methodology/Principal Findings:**

Here, we used *kakusei* to analyze the relationship between MB neuron activity and types of foraging behavior. The number of *kakusei*-positive MB neurons was higher in the round dancers that had flown a short distance than in the waggle dancers that had flown a long distance. Furthermore, the amount of *kakusei* transcript in the MBs inversely related to the waggle-phase duration of the waggle dance, which correlates with the flight distance. Using a narrow tunnel whose inside was vertically or axially lined, we manipulated the pattern of visual input, which is received by the foragers during flight, and analysed *kakusei* expression. The amount of *kakusei* transcript in the MBs was related to the foraging frequency but not to the tunnel pattern. In contrast, the number of *kakusei*-positive MB neurons was affected by the tunnel patterns, but not related to foraging frequency.

**Conclusions/Significance:**

These results suggest that the MB neuron activity depends on the foraging frequency, whereas the number of active MB neurons is related to the pattern of visual input received during foraging flight. Our results suggest that the foraging frequency and visual experience during foraging are associated with different MB neural activities.

## Introduction

The honeybee (*Apis mellifera* L.) is a eusocial insect with a highly ordered society. Foragers that find a rich food source return to the hive and transmit information about the location of the food source by ‘dance communication’ [1], [2], [3], [4]. They perform a ‘round dance’ when the food source is a very short distance away (less than approximately 50 m) and a ‘waggle dance’ when the food source is a long distance away (>50 m) [2], [3]. In the waggle dance, the ‘distance’ and ‘direction’ of the food source are encoded as the ‘duration’ and ‘angle’ of the waggle-phase of the dance, respectively. Other nestmate foragers then decode the dance into spatial information about the food source, which allows them to access the same food source [2], [3], [5].

Recent studies revealed that foragers estimate flight distance using a visually-driven ‘odometer’ based on the amount of ‘optic flow’ (the flow of visual information that crossed the visual field) that they perceive during the foraging flight [6], [7]. Flight direction, on the other hand, is estimated using the sun as a compass [2]. The sensory information perceived during foraging must be integrated and encoded in the brain before the dance is expressed in the hive. The neural mechanisms underlying the integration of the sensory information, however, were not understood.

We previously established a method to visualize neural activity in the honeybee brain, using a newly discovered immediate early gene, which we termed *kakusei* [8]. *kakusei*-expression was induced transiently in the brains of workers with a peak at 30–60 min after seizure induced by awakening the workers from anesthesia, and was useful to detect neural activity in the worker brains. In addition, *kakusei*-transcript contained no significant open reading frame that encodes a protein, and was located in the nuclei of the worker brains, suggesting that *kakusei*-transcript functions as a non-coding nuclear RNA [8]. In the forager brains, *kakusei* was preferentially expressed in a mushroom body (MB) neuron subtype, the small-type Kenyon cells (sKCs) [8]. In contrast, the sKC-preferential expression pattern of *kakusei* was not observed in the other behavioral groups, including the nurse bees, light-exposed bees (workers that had been exposed to white light after dark adaptation [8]), and bees that underwent the re-orienting flights after the hive was moved. These results strongly suggested that the sKC-preferential neural activity is related specifically to the behavioral components of foraging, rather than to common behavioral traits among foraging and other behaviors, such as the flying experience and/or memorizing landmarks.

**Figure 1:**
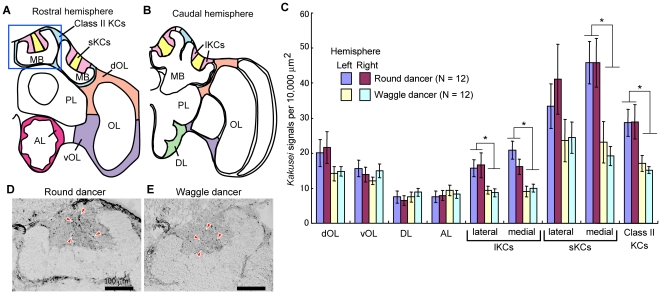
Round dancers have a greater number of active MB neurons than the waggle dancers. (A and B) Schematic drawings of the rostral (A) and caudal (B) hemispheres of the worker honeybee brain. The brain regions in which signals were counted are shown in different colors. (C) Quantification data of *kakusei*-signal densities in various brain regions of the round and waggle dancers. Expression of *kakusei* was detected by *in situ* hybridization using coronal brain sections. A significant difference in *kakusei* expression between the dancers was detected in the MB neurons. There was no significant difference between the right and left hemispheres for any brain region (*P*>0.05). All data are represented as the mean ± standard error. Asterisks indicate *P*<0.05 calculated by the two-factor ANOVA. (D and E) Representative images of *in situ* hybridization of *kakusei* in the calyces of the MBs of the round and waggle dancers, respectively. The corresponding brain region is shown as a blue square in A. Some of the *kakusei* signals are indicated by red arrowheads. Scale bars: 100 µm. Abbreviations: AL, antennal lobe; Class II KCs, class II Kenyon cells; DL, dorsal lobe; dOL, dorsal OL; lKCs, large-type Kenyon cells MB, mushroom body; OL, optic lobe; PL, protocerebral lobe; ; sKCs, small-type Kenyon cells; vOL, ventral OL.

The MBs of the honeybees are a paired brain structure that comprise two cup-like structures called the lateral and medial calyces (Fig. 1A and B). Each calyx contains the sKCs, the large-type KCs (lKCs), and the class II KCs. The sKCs are located on the center of each calyx and receive various sensory information at the basal ring zone of the calyx, whereas the lKCs are located on the inside edge of each calyx and receive olfactory and visual information at the lip and collar zones of the calyx, respectively [9]. The class II KCs are located around the calyces, receive inputs from the entire calyx, and compose the γ lobe [9], [10]. In spite of these anatomical data, the sensory information and neural mechanisms that induce the sKC-preferential neural activity in the forager brain is still elusive.

In the present study, to elucidate the sensory basis that underlies the MB neuron activity in the forager brain, we investigated the relationship between *kakusei* expression and the types of foraging behavior. Through analyses of the round and waggle dancers, we observed a higher number of MB neurons are active in the round dancers than in the waggle dancers. In addition, through the analysis of foragers that flew through the narrow tunnels with vertical or axial stripes, we observed that the amount of *kakusei* transcript correlated with the foraging frequency, while the *kakusei*-signal density is different between the tunnel patterns. Our results suggest that the foraging frequency and visual experience during the foraging flight are associated with the different MB neural activities in the forager brains.

## Results

### Round dancers have a higher number of *kakusei*-positive MB neurons than waggle dancers

To examine whether the type of foraging behavior is related to neural activity in the forager brain, we first compared the number of *kakusei*-positive neurons in the brain of the round and waggle dancers, which visited food sources at short and long distances, respectively. To collect the round dancers, we trained marked foragers to visit a feeder that was set near the observation hive (approximately 5 m from the hive). We collected marked foragers that performed the round dances in the observation hive as ‘round dancers’. We also collected foragers that performed waggle dances in the same observation hive as ‘waggle dancers’, which indicated that they had visited natural food sources that are located more than 50 m far from the hive.

*In situ* hybridization of *kakusei* was performed to detect active neurons in the brains of the round and waggle dancers (N = 12, respectively). *kakusei* encodes a nuclear non-coding RNA and localizes exclusively in the nuclei, and thus the number of *kakusei*-signals can be counted and quantified [8]. The sKC-preferential *kakusei*-expression pattern was observed in both the round and waggle dancers, consistent with our previous report (Fig. 1D and E) [8]. Signal-count and quantification was performed for each brain region shown in Fig. 1A and B. In some MB neurons (the lKCs in both calyces, the sKCs in the medial calyx, and the class II KCs), the *kakusei*-positive neuron density (number of *kakusei*-signals/10,000 µm^2^) was significantly higher in the round dancers than in the waggle dancers [Fig. 1C, *P*<0.05, two-factor analysis of variance (ANOVA; F1: dance type; F2: brain hemisphere)]. The proportion of *kakusei*-positive cells among sKCs compared to that among lKCs was 2.8±0.25 for the round dancers and 2.2±0.21 for the waggle dancers. In our previous study [8], re-orienting flight induced *kakusei* expression in all KC types and the proportion was approximately 1.5 after a 30-min re-orienting flight. Thus, the present results again indicate that the sKC-preferential *kakusei*-expression is not due to the difference in the cell density between sKCs and lKCs, but rather to the preferential activation of sKCs. There was no significant difference in *kakusei*-signal density between the right and left brain hemispheres in any brain region (Fig. 1C). These findings indicate that a higher number of MB neurons are active in the round dancers than in the waggle dancers, strongly suggesting that differences in the foraging distance are reflected as differences in the number of active MB neurons in the forager brain.

### The amount of *kakusei* transcript in the MBs of the waggle dancers was inversely correlated with the waggle-phase duration of the dance

**Figure 2:**
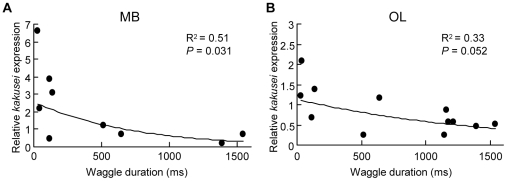
The amount of *kakusei* transcript in the brains of waggle dancers inversely correlates with the waggle-phase duration. The relative amount of *kakusei* transcript was quantified by real-time RT-PCR and plotted against the waggle-phase duration. The MBs (A) and OLs (B) are indicated. The logarithmic regression lines are shown. The *R^2^* and *P* values for the regression lines are shown. Note that the slope of the MBs is steeper than that of the OLs.

To more closely investigate whether the foraging distance is related to MB neural activity in the forager brain, we analysed the relationship between the amount of *kakusei* transcript and the waggle-phase duration of the waggle dances. The relative amount of *kakusei* transcript in the MBs and optic lobes (OLs) was quantified by real-time reverse transcription-polymerase chain reaction (RT-PCR) and plotted against the waggle-phase duration of the dances, which were video-recorded and analysed using a transparent observation hive (Fig. 2). An inverse relation was observed between the amount of *kakusei* transcript and the waggle-phase duration in the MBs (Fig. 2A, *P*<0.05). An especially high level of *kakusei* transcript was detected in the waggle dancers whose waggle duration was less than approximately 150 ms. On the other hand, there was no significant relationship between the amount of *kakusei* transcript and the waggle-phase duration in the OLs, although there was a similar tendency for the amount of *kakusei* transcript was higher in foragers that flown short distance (Fig. 2B). These results indicated that the ‘distance’ of the foraging flight was also inversely related to the extent of neural activity in the MBs. We also conducted the same analysis between the amount of *kakusei* transcript and the angle of the waggle-phase, to examine whether the ‘direction’ of the foraging flight is related to the neural activity in the forager brain, but we observed no significant relation between them (regression analysis: *P* = 0.69 and 0.93 for MBs and OLs, respectively).

To further examine whether the *kakusei*-signal density in the MBs is related to the waggle-phase duration, we investigated *kakusei* expression by *in situ* hybridization, calculated the *kakusei*-signal density for each KC type, and plotted them against the waggle-phase duration (Fig. S1). In this experiment, we only could catch the foragers whose waggle-phase durations were more than 650 ms. There was no significant relationship between the *kakusei*-signal density and the waggle-phase duration in any brain region of the MBs. These results indicate that the number of active MB neurons is not related to the waggle-phase duration that was more than 650 ms, which was again consistent with the fact that the *kakusei*-signal density was higher in round dancers than waggle dancers (Fig. 1). These results are also consistent with the finding that a strong association between waggle-phase duration and the amount of *kakusei* transcript was detected only in short waggle-phase duration (<150 ms) (Fig. 2). We also investigated the relationship between *kakusei*-signal density and the angle of the waggle-phase, and observed no significant relationship (*P*>0.1 each: regression analysis). These results suggest that the direction of the foraging flight is not reflected to the *kakusei* expression in the brains of waggle dancers.

### The amount of *kakusei* transcript in the MBs correlated with the foraging frequency in tunnel experiment

We propose three possible reasons for the increased neural activity in the MBs observed in the above experiments (Figs. 1 and 2), as follows. First, the MB neural activity may be induced depending on the frequency of visits to the food source. Under free flight, we did not assess how often short distance foragers with high gene expression returned to the hive. It is therefore conceivable that the shorter the flight the more often foragers fly. The repeated foraging may induce a high MB neural activity, which might be reflected to a higher level of *kakusei* expression. Second, there may be some neural mechanisms that induce a higher level of neural activity when the distance to the food source is short. Third, the amount of *kakusei* transcript decreases when the distance of the foraging flight increases, due to desensitization to continuous visual input.

To evaluate these possibilities, we investigated the amount of *kakusei* transcript in the brains of foragers whose distance sense was artificially controlled. The distance sense of the honeybee forager depends on the amount of the optic flow received during the foraging flight [6], [11]. Thus, we trained the individually marked foragers to fly though a narrow tunnel whose inner walls and floor were lined either vertically or axially. The flight through the vertically-lined tunnel increases the amount of optic flow, compared with the flight through the axially-lined tunnel [12]. After training, we allowed the foragers to freely visit the feeder and video-recorded their behavior for 1 h, because *kakusei* expression lasts from 30 min to 1 h after neural activity [8].

**Figure 3:**
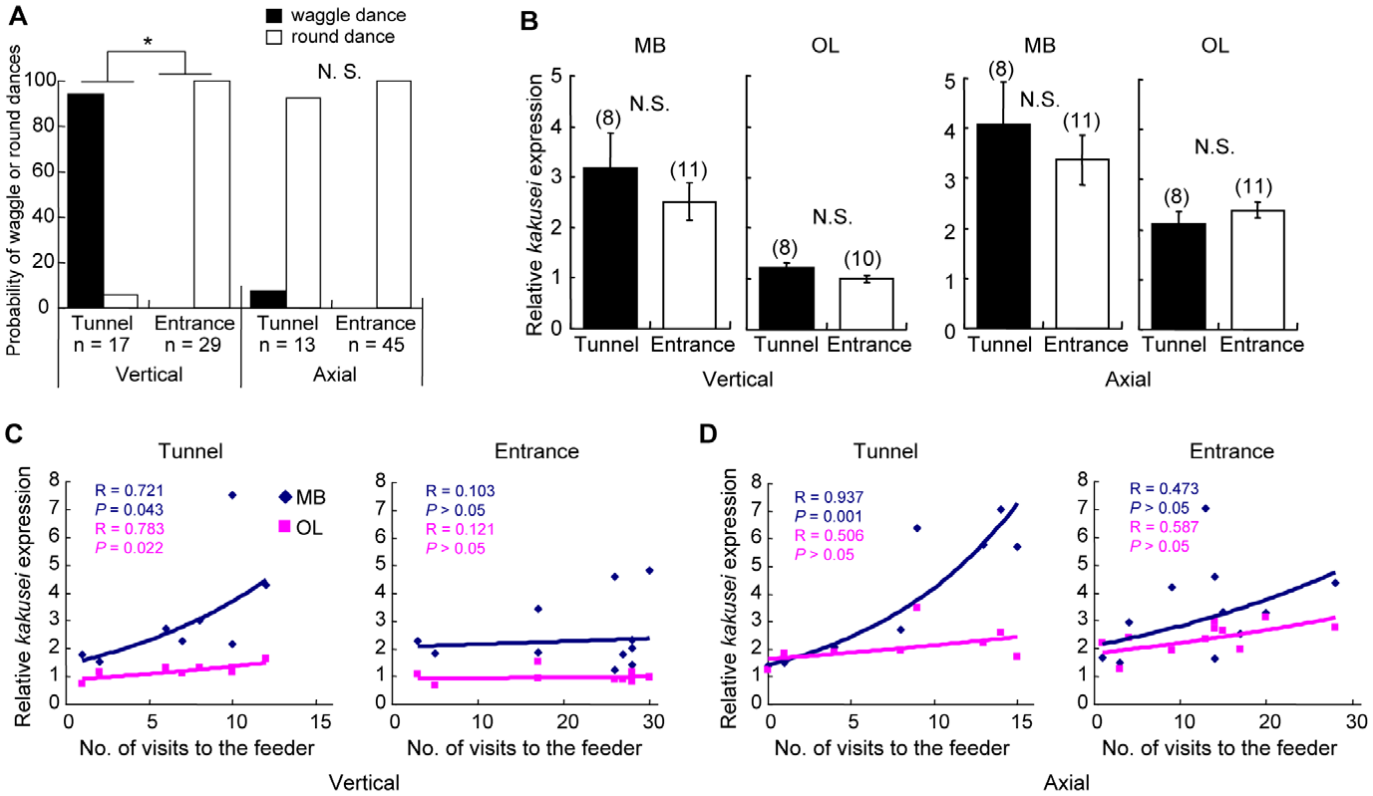
The amount of *kakusei* transcript in the brain correlates to the foraging frequency of the foragers. (A) The probabilities of waggle or round dances. The left panel shows the data from the vertically-lined tunnel, and right panel shows the data from the axially-lined tunnel. In each tunnel experiment, the dance probabilities were compared between the tunnel and entrance groups. n indicates the number of dances analyzed. *: *P*<0.0001, χ^2^ test. N.S.: not significant. (B) The relative amount of *kakusei* transcript was quantified by real-time RT-PCR and compared between the tunnel and entrance groups. Left and right panels show data from the vertically- and axially-lined tunnels, respectively. Data from the MBs and OLs are shown separately. (C) and (D) The relative amount of *kakusei* transcript was plotted against the number of visits to the feeder during 1 hr. Data from the vertically-lined tunnels (C) and axially-lined tunnels (D) are shown. The tunnel and entrance groups were shown left and right panels, respectively. Note the robust increase in *kakusei* expression in the MBs. The *R* and *P* values for each regression line are shown.

During the 1–h observation, 94% (16/17) of the foragers that returned from the feeder placed 8 m inside the vertically-lined tunnel (tunnel group) performed the waggle dance in the hive (Fig. 3A). The average waggle-phase duration was 209.52±14.03 ms [N = 70 (waggle-phases), n = 16 (waggle dances)]. When the feeder was placed at the entrance of the vertically-lined tunnel (entrance group), every dance was a round dance (n = 29) (Fig. 3A). A significant difference in dance behavior was observed between the tunnel and entrance groups (*P*<0.0001, χ^2^ test). In contrast, only 8% (1/13) of the foragers that flew through the axially-lined tunnel performed the waggle dance, and the remaining 92% performed the round dance (n = 13) (Fig. 3A). In the entrance group, every bee performed the round dance (n = 45) (Fig. 3A). No significant difference in dance behavior was observed between the tunnel and entrance groups (*P*>0.05, χ^2^ test). These results indicate that we reliably manipulated the distance sense of the foragers.

Using these foragers trained in the vertically- or axially-trained tunnels, we quantified the relative amount of *kakusei* transcript in the MBs and OLs by real-time RT-PCR (Fig. 3B). No significant difference in the level of *kakusei* expression was observed between the tunnel and entrance groups in either the MBs or OLs, irrespective of the tunnel patterns. These results strongly suggest that the amount of optic flow that the foragers perceived during the foraging flight was not reflected to the amount of *kakusei* transcript in the forager brain, arguing against the second and third possible explanations stated above.

Next, to investigate whether the foraging frequency has effects on the extent of neural activity, we plotted the relative amount of *kakusei* transcript against the foraging frequency (number of visits to the feeder during the 1-h observation). A statistically significant relationship was observed in the MBs of both tunnel groups and in the OLs of the vertically-lined tunnel group (Fig. 3C and D). Their relationships were best fitted by a logarithmic function (statistical values are shown as insets). The slopes of the regression lines were steeper in the MBs than in the OLs in both tunnel groups, indicating that *kakusei* expression in the MBs was more responsive to foraging frequency than that in the OLs. In contrast, we observed no definitive correlation between the amount of *kakusei* transcript and foraging frequency in the entrance groups (Fig. 3C and D). In these experiments, the distribution of the number of visits was different between the tunnel and entrance groups. This bias was unavoidable, because the tunnel entrance was easy for foragers to visit whereas the tunnel was not. Even when we analyzed the foragers with fewer than 15 visits, however, there were no statistical differences. These results support the first explanation stated above that the amount of *kakusei* transcript in the MBs depends on the frequency of the visits to the food source, provided that the foragers flew through the tunnels.

### Foragers that flew in the vertically-patterned tunnel had greater *kakusei*-signal densities in the MB neurons than foragers that flew in the axially-patterned tunnel

Since it was suggested that the factor that increases *kakusei* expression in the forager MBs is not the amount of optic flow, but the foraging frequency, we next examined whether this is also the case for the *kakusei*-signal density. We conducted *in situ* hybridization and investigated the *kakusei*-signal densities in the MB neurons of the foragers that flew in the vertically- or axially-lined tunnels. The foragers that visited the entrance feeder were not analysed, because any significant relationship was not observed between the amount of *kakusei* transcript and foraging frequency for these foragers in the previous experiments (Fig. 3). These tunnel experiments were performed independent of the above tunnel experiments, and we again confirmed that the flight through the vertically-lined tunnel significantly increased the probability of the waggle dance, while the flight though the axially-lined tunnel did not (data not shown).

**Figure 4:**
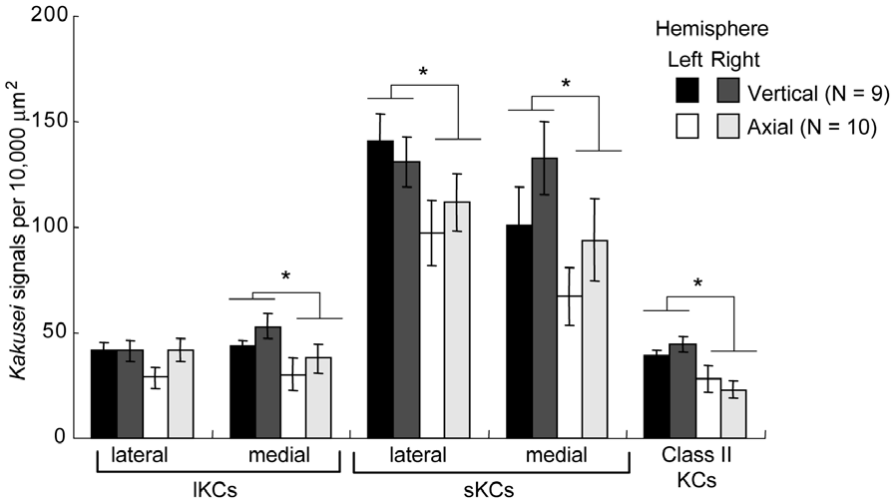
A greater number of MB neurons were *kakusei*-positive in the foragers that flew in the vertically-lined tunnel than in those that flew the axially-lined tunnel. The *kakusei*-signal densities in the MBs were compared between foragers that flew in the differently-patterned tunnels. *: *P*<0.05, two-factor ANOVA.

In contrast to our expectation, significantly higher *kakusei*-signal densities were detected in almost all MB regions, except the lateral lKCs, of foragers that flew the vertical-pattern tunnel than in those that flew the axial-pattern tunnel [Fig. 4: *P*<0.05, two-factor ANOVA (F1: tunnel pattern; F2: brain hemisphere)]. No significant difference was observed between the right and left hemispheres (*P*>0.05). We also investigated the relationship between the *kakusei*-signal density in each MB region and the foraging frequency. No statistically significant relationship, however, was observed in any MB region with either tunnel pattern (Fig. S2). Rather, there was a tendency in almost all MB regions, except lKCs and sKCs of foragers that have flown thorough tunnel with vertical strips, that the *kakusei*-signal density decreased as the foraging frequency increased, which is opposite to the relationship between the amount of *kakuse*i transcript and foraging frequency (Fig. 3C and D). These results suggest that the number of active MB neurons is not related to foraging frequency, but to the amount of optic flow received during foraging flights. Thus, these results implies differential effects of the amount of optic flow received during foraging flights and the foraging frequency on the *kakusei* expression in the MBs in the tunnel experiments: while the former was related to the *kakusei*-signal densities, the latter was related to the amount of *kakusei* transcript in the MBs.

## Discussion

In the present study, we investigated the relationship between foraging behavior and neural activity in forager brains using the neural activity marker *kakusei*. Here we evaluated neural activity based on two factors, the amount of *kakusei* transcript and the *kakusei*-signal density, as indicators of the total amount of neural activity and the number of active neurons, respectively. The ‘distance’-related behavioral component, which was expressed by the dance type (Fig. 1) or the waggle-phase duration (Fig. 2), was strongly related to *kakusei* expression in the MBs. The tunnel experiments further revealed that the amount of *kakusei* transcript was related to the frequency of foraging through the tunnel (Fig. 3), whereas the number of *kakusei*-positive neurons was related to the tunnel pattern (Fig. 4). The present study is the first to report how dance type and flight parameter are associated with MB neuron activity in forager honeybees.

### Foraging frequency-dependent increase in the amount of *kakusei* transcript in the MBs

In the present study, the waggle-phase duration was inversely related with the amount of *kakusei* transcript in the MBs (Fig. 2A). In addition, foraging frequency was positively related to the amount of *kakusei* transcript in the MBs when the foragers flew in the tunnel (Fig. 3C and D). Workers that visit a nearer food source can repeat foraging more frequently. Thus, these results suggest that neural activity in the MBs tended to occur in a foraging frequency-dependent manner. In contrast, there was no significant relationship between foraging frequency and the amount of *kakusei* transcript in the MBs when foragers visited the tunnel entrance (Fig. 3C and D). Why was this significant relationship observed only when foragers flew the tunnel? One explanation is that the foraging flight through the tunnel induces higher neural activity in the MB neurons than flights to the tunnel entrance. The foragers use many cues during foraging [3] and the tunnel flight demands higher integration of visual information [13]. Thus, it is possible that more complex information processing is required for the tunnel flight than for the visit to the entrance, and was reflected as the foraging frequency-dependent increase in *kakusei* expression only in the tunnel flight.

The relationship between the foraging frequency and the amount of *kakusei* transcript was closer in the MBs than in the OLs (Fig. 3C and D). These results suggest that the MB neurons are more sensitive to the sensory information perceived during the repetitive foraging flight than the OL neurons. The MBs are considered to be a higher center of the insect brain and a variety of sensory modalities project to the MBs [14], [15]. In the honeybee, information from all of the sensory modalities investigated so far, including olfactory, visual, mechanosensory, and gustatory information, is transmitted to the MBs [9], [16], [17]. From the viewpoint that the MBs function as an integrator of multimodal sensory information, the present results corroborate earlier assumptions that more than mere sensory processing, like integration of foraging information, occurs in the MBs of the foragers.

### Dance type- and tunnel pattern-dependent changes in the *kakusei*-signal density in the MBs

At the beginning of this study, we found that the *kakusei*-signal density in the MBs was higher in the round dancers than in the waggle dancers in a field experiment (Fig. 1). These findings indicate that a greater number of MB neurons was active in the round dancers than in the waggle dancers in the field experiments. In contrast, the *kakusei*-signal density in the MBs was higher in foragers that flew the vertically-lined tunnel (waggle dancers) than in those that flew the axially-lined tunnel (round dancers) (Fig. 4). These finding indicates that the number of active MB neurons was higher in the waggle dancers than in the round dancers in the tunnel experiments; therefore, opposing results were observed in the field experiments. The discrepancy between these experiments implies that *kakusei*-signal density in the MBs is not a simple reflection of the foraging flight parameters, but rather varies depending on the experimental conditions. This variability might be one of the reasons that we could not detect a significant relationship between the *kakusei*-signal density in the MBs and the distance-related behavioral components (waggle-phase duration or foraging frequency) in either the field or tunnel experiments (Figs. S1 and S2). Although future experiments for verification under controlled conditions are necessary, we speculate at present that multiple factors, including the amount of the optic flow and foraging frequency, affect *kakusei*-signal density in the MBs. Otherwise, it should be considered that the tunnel flight (8 m) only simulates short flights, expressed as a waggle-phase duration of approximately 200 ms. This represents the lowest values in Fig. 2, and therefore not all aspects of free flight can be modeled by the tunnel experiments.

In the tunnel experiments, a significant relationship was detected between the amount of *kakusei* transcript and foraging frequency (Fig. 3), but not between the *kakusei*-signal density and foraging frequency (Fig. S2). These findings suggest that the foraging frequency-dependent increase in the *kakusei* transcript occurs without an increase in the number of *kakusei*-positive neurons. With regard to this issue, we speculate that the visual information received during foraging is encoded in an ensemble of MB neurons, and that repetitive foraging stimulates the same set of neurons repeatedly, resulting in an increase in the amount of *kakusei* transcript in each neuron. Future experiments to evaluate this hypothesis are necessary.

It is plausible that the increased *kakusei*-signal density detected in the MBs of the foragers that flew vertically-lined tunnel reflects a change in the amount of or pattern of visual inputs to the MBs. Recently, we showed that neural activities of both the gamma-aminobutyric acid-positive and negative neurons are increased in the OLs of the foragers, suggesting that complex visual processing occurs in the OLs in the forager brain [18]. Thus, it is possible that changes in the visual inputs during foraging flight are sensitively reflected by the activity pattern in both the OLs and MBs in the forager brain. A future study in which MB function is impaired will reveal the functional importance of the MB neurons in visual information processing and provide important clues to elucidate the neural mechanisms of dance communication.

## Materials and Methods

### Bees

European honeybees (*A. mellifera* L.) were purchased from a local dealer (Kumagaya Honeybee Farm, Saitama, Japan) and maintained at the University of Tokyo. Experimental colonies were housed in two-frame transparent observation hives containing approximately 4000 bees. Each honeybee was caught immediately after the dance was visually confirmed (waggle or round) or the dance behavior was video-recorded, respectively. The bees caught from the observation hives were immediately anesthetized with ice-cold water and kept on ice until use for *in situ* hybridization or real-time RT-PCR to maintain the current state of the *kakusei* transcript in the brain.

### Behavioral observation and data analysis

Dances performed on the vertical comb of the observation hives were recorded using a digital video camera (HDR-HC3; SONY, Japan) by setting the camera in front of the observation hive. The recording rate was 30 frames/s. The video data were analysed using Move-tr/2D software (Library, Tokyo, Japan). Waggle-phase duration was measured using units of video frames and thus calculated by multiplying the number of frame units by 1/30 s. A waggle-phase was defined as the portion of the dance period during which the dancer made waggle movements. The angle of the waggle-phase was measured as a clockwise angle of the waggle run to the vertical axis of the hive. Waggle-phase duration and the angle of each dance of the bees were measured repeatedly and the averaged values for each bee were analysed.

### Tunnel and bee training

In the tunnel experiments, to ensure that the all foragers visited the feeder, the observation hive and the tunnel were enclosed in insect screen cloth. The tunnel, which was made of 5-mm thick transparent plastic, was 11 cm wide, 20 cm high, and 8 m long. The sidewalls and floor of the tunnel were lined with black and white vertical stripes at 3.6-cm intervals or axial stripes at 8-cm intervals, according to previous studies [6], [7], [12], [19]. The patterns were generated by a computer, printed on a laser printer, and affixed to the walls using transparent mending tape [19]. The tunnel was placed 2 m away from the observation hive, with the entrance facing the hive entrance, and the far-end kept closed. The tunnel was also covered with transparent plastic plates to allow foragers to utilize the sun as a compass. Each forager was individually marked on its dorsal thorax and abdomen using colored markers in the morning [2], [4]. The marked foragers were trained to fly to a feeder containing sugar solution (1 M, non-scented) placed at the far-end of the tunnel. First, the feeder was placed near the observation hive. After a sufficient number of foragers were attracted, the feeder was gradually and progressively moved to further inside the tunnel. After the feeder reached the far-end of the tunnel (8 m away from the entrance), video-recording of both the feeder and the observation hive was started. After 1 h of video-recording, the foragers were caught at the feeder and used for *kakusei*-expression analysis. In each experiment, training and sampling were conducted with the pair of tunnel and entrance groups on the same day. The experiments with the different pattern tunnels, however, were conducted on different days. Therefore, the data were analysed by the experimental day. Experiments for each batch (for each Fig. data) were conducted on successive days using the same hive.

### *In situ* hybridization and image analysis

*In situ* hybridization was performed as described previously [8]. Frozen coronal brain sections (10-µm thick) were fixed in 4% paraformaldehyde in phosphate-buffered saline, pretreated, and hybridized with digoxigenin (DIG)-labeled riboprobes as previously [8], [18]. The DIG-labeled riboprobes were synthesized by T7 or SP6 polymerase with a DIG labeling mix (Roche) from a template containing the fragment from +4511 to +5159. After several stringent washes, DIG-labeled riboprobes were detected immunocytochemically with peroxidase-conjugated anti-DIG antibody (1∶500; Roche) and a TSA Biotin System (Perkin Elmer). Sense probes were used as negative controls and the signals were confirmed to be antisense probe-specific. The experimental procedures lead to differences in the conditions of the color reaction and signal development among experiment batches (see differences between Figs. 1 and 4). Thus, experiments, quantification, and comparison were conducted with each batch.

For quantification, the brain regions to be analysed were defined as shown in Fig. 1A and B, as previously described [8]. Each brain region (specific soma area) was selected using a Pen Tablet (Wacom) and the area was measured using ImageJ analysis software (NIH, http://rsb.info.nih.gov/ij). At the same time, the number of *kakusei*-signals in the selected area was manually counted and divided by the area to calculate the *kakusei*-signal density. To reduce the deviation of signal densities between sections, signal-count and signal density calculation were performed using two sections which include brain regions of interest (Fig. 1A and B) for each individual, and the average value was used for analysis. The density of *kakusei*-signals was presented as the value relative to 10,000 µm^2^. Micrographs were numbered and signals were counted by an investigator blind to the test group. Statistical analyses were conducted using JMP (SAS) and Excel (Microsoft) software. Data are shown means ± standard error (SEM). The *kakusei*-signal density ratio between the sKCs and lKCs was calculated by dividing the *kakusei*-signal density in the sKCs by that in the lKCs.

### Quantification of *kakusei* transcript

The relative amount of *kakusei* transcript was quantified by real-time RT-PCR. Each forager whose dances were video-recorded was anesthetized on ice and the brain was dissected out of the head under a microscope. The brain was then manually divided into the MBs, OLs, and remaining other brain regions using fine forceps. Because *kakusei* expression was under the detection threshold in the remaining brain regions, analysis was conducted only in the MBs and OLs. Total RNA was extracted from each brain region of the individual bees using TRIzol (Invitrogen). The isolated total RNA was treated with DNase I (Invitrogen), and reverse-transcribed with SuperScript III (Invitrogen). Real-time PCR was performed with Light Cycler-DNA master hybridization probes (Roche) according to the manufacturer's protocol using gene-specific primers (*kakusei*; 5′-GGAAACAGGTGGTTTGATGACCATTG and 5′-CACGTTCCAAGGTTTAACGATGCG, *actin*; 5′-GAAATGGCAACTGCTGCATC and 5′-TCCACATCTGTTGGAAGGTG) and fluorescent probes (*kakusei*; fluorescein isothiocyanate (FITC) probe, 5′-CGCTGTAGTGCGTTTTCACTCGGATCGA, and LC-Red640 probe, 5′-TCCGAGGAAATCCGAGCAAAGTTCGTTC, *actin*; FITC probe, 5′-CCATGAAAATTAAGATCATCGCGCCAC, and LC-Red640 probe, 5′-CGAGAAGAAATATTCCGTATGGATTGGTG). The relative amounts of *kakusei* and *actin* were determined using the quantification standards. PCR products amplified by *kakusei* or *actin* primer pairs were purified using a PCR purification kit (QIAGEN), serially diluted (10-fold dilutions for a dynamic range of 10^6^), and used as quantification standards. The determined amount of *kakusei* transcript was divided by that of *actin*, and is shown as the relative level of *kakusei* expression. Data under the detection threshold (outside of the quantification standard range) were excluded from the analysis.

## Supporting Information

Figure S1 **Relationship between the** ***kakusei*** **-signal densities and the waggle-phase duration.** There was no significant correlation between the number of *kakusei*-positive cells and the waggle-phase duration (*P*>0.05). The data from the left and right hemispheres are shown in the upper and lower panels, respectively. Each panel shows data from each brain region, the lKCs, sKCs, and class II KCs. Data obtained from the lateral and medial calyces are shown in different colors.
(TIF)

Figure S2 **Relationship between the** ***kakusei*** **-signal densities and the number of visits to the feeder.** Data from the left and right hemispheres were shown in (A) and (B), respectively. There was no obvious correlation between the *kakusei*-signal densities and the number of feeder visits in either the vertically-lined tunnel (upper panels) or the axially-lined tunnel (lower panels).
(TIF)
