# Supplementary figures and images for: Dance Type and Flight Parameters Are Associated with Different Mushroom Body Neural Activities in Worker Honeybee Brains
Source: PLoS One. 2011 Apr 26;6(4):e19301. doi: 10.1371/journal.pone.0019301 (PMC3082565; doi:10.1371/journal.pone.0019301)

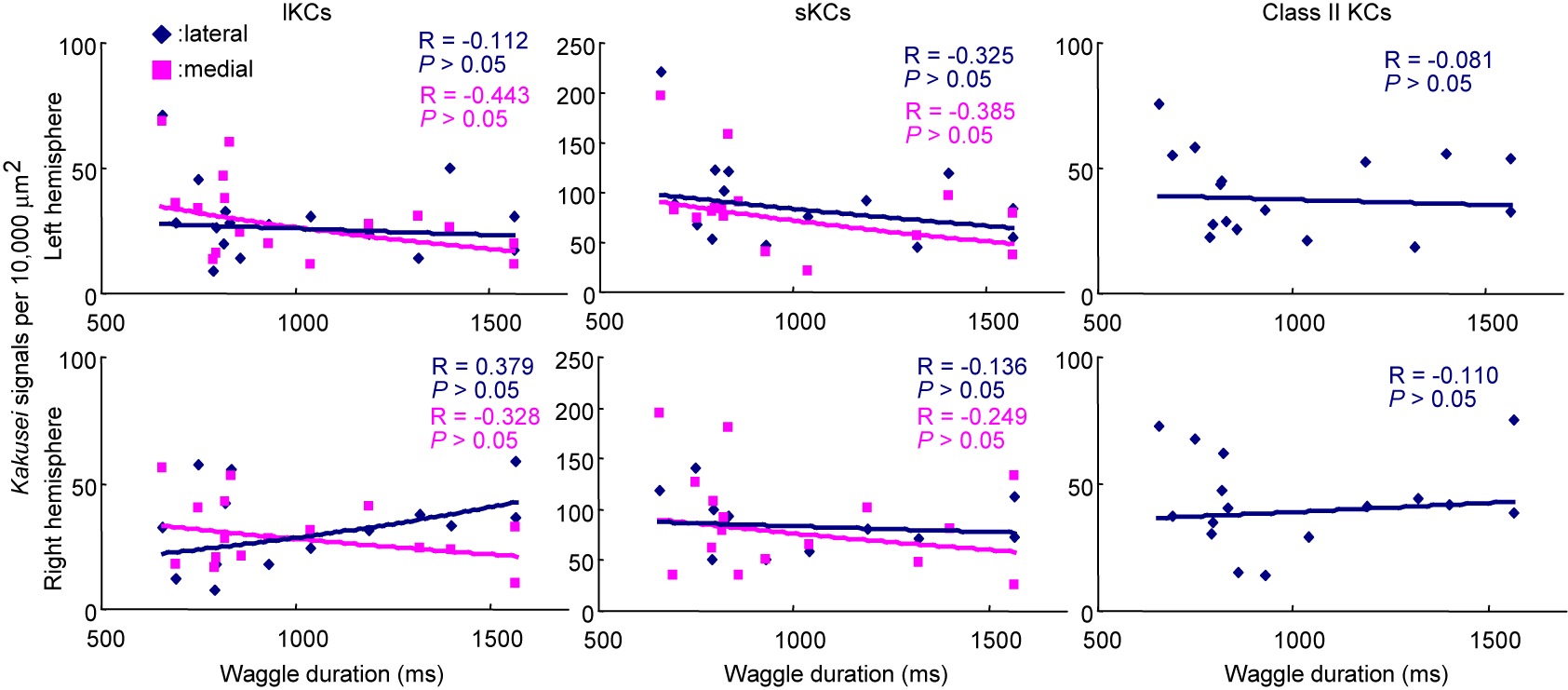

Supplement: Figure S1 — Relationship between the kakusei -signal densities and the waggle-phase duration. There was no significant correlation between the number of kakusei-positive cells and the waggle-phase duration (P>0.05). The data from the left and right hemispheres are shown in the upper and lower panels, respectively. Each panel shows data from each brain region, the lKCs, sKCs, and class II KCs. Data obtained from the lateral and medial calyces are shown in different colors. (TIF) [file pone.0019301.s001.tif]

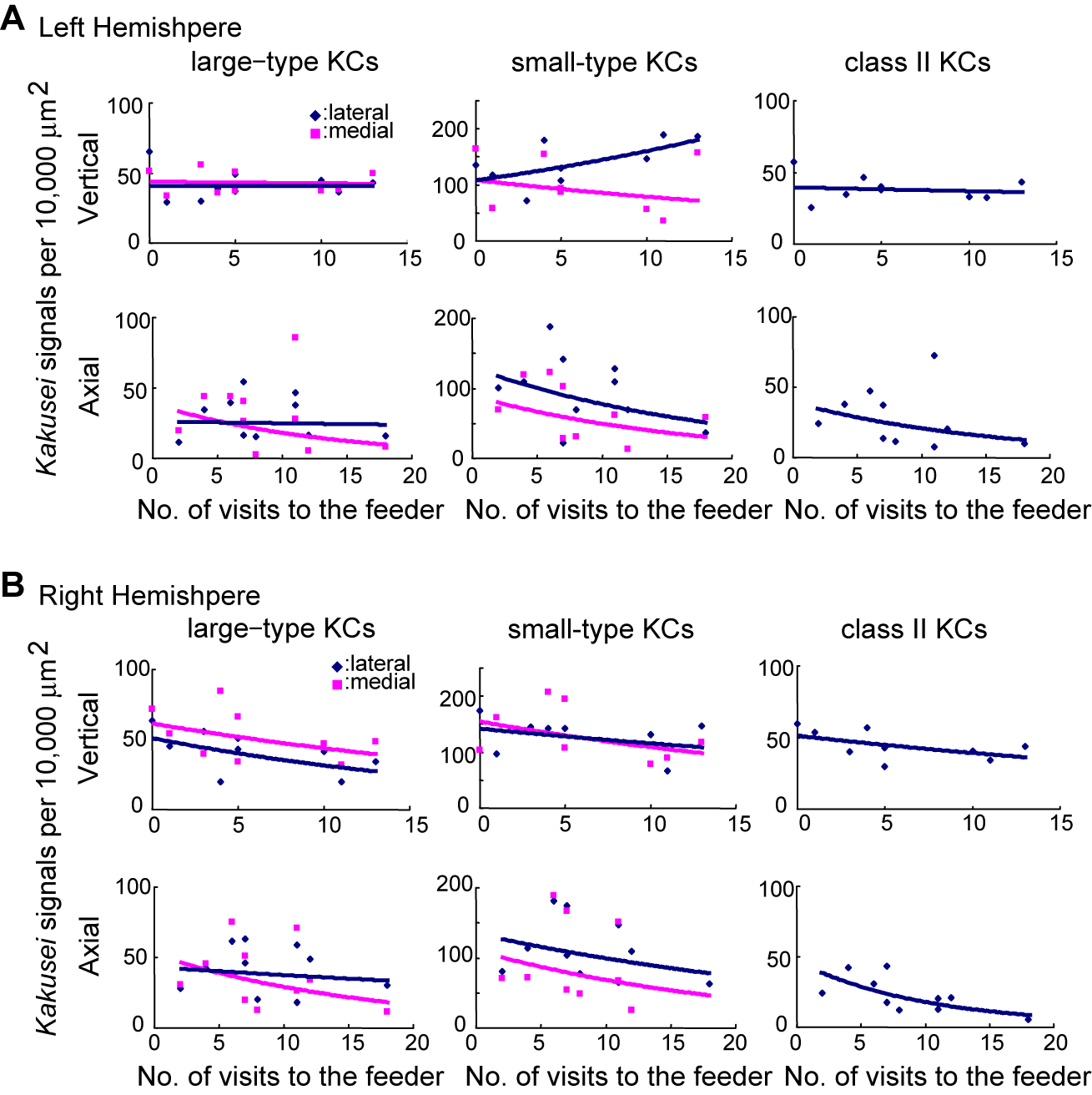

Supplement: Figure S2 — Relationship between the kakusei -signal densities and the number of visits to the feeder. Data from the left and right hemispheres were shown in (A) and (B), respectively. There was no obvious correlation between the kakusei-signal densities and the number of feeder visits in either the vertically-lined tunnel (upper panels) or the axially-lined tunnel (lower panels). (TIF) [file pone.0019301.s002.tif]
